# A decade of modelling research yields considerable evidence for the importance of concurrency: a response to Sawers and Stillwaggon

**DOI:** 10.1186/1758-2652-14-12

**Published:** 2011-03-15

**Authors:** Steven M Goodreau

**Affiliations:** 1 Department of Anthropology, University of Washington, WA, 98195 USA

## Abstract

In their recent article, Sawers and Stillwaggon critique the "concurrency hypothesis" on a number of grounds. In this commentary, I focus on one thread of their argument, pertaining to the evidence derived from modelling work. Their analysis focused on the foundational papers of Morris and Kretzschmar; here, I explore the research that has been conducted since then, which Sawers and Stillwaggon leave out of their review. I explain the methodological limitations that kept progress on the topic slow at first, and the various forms of methodological development that were pursued to overcome these. I then highlight recent modelling work that addresses the various limitations Sawers and Stillwaggon outline in their article. Collectively, this line of research provides considerable support for the modelling aspects of the concurrency hypothesis, and renders their critique of the literature incomplete and obsolete. It also makes clear that their call for "an end (or at least a moratorium) to research on sexual behaviour in Africa" that pertains to concurrency is unjustified.

## Introduction

In their recent article in this journal [1], Sawers and Stillwaggon critique the "concurrency hypothesis" on a number of grounds. They argue that neither the mathematical modelling work nor the behavioural data provide a convincing picture for the importance of relational concurrency in explaining national or regional disparities in HIV burden. They then call for "an end (or at least a moratorium) to research on sexual behaviour in Africa of the kind discussed in this article" (that is, either modelling or empirical studies on relational concurrency).

In this paper I focus on one thread of their argument, that pertaining to the modelling work. Sawers and Stillwaggon focused their entire critique of the modelling literature on Morris and Kretzschmar's initial proof-of-concept papers [2-4]. Although these may be the most widely cited on the topic because of their foundational role, they are not the entire field. Sawers and Stillwaggon essentially argue that since these initial papers made some unrealistic assumptions, the entire line of research should be ended. From this argument, the general reader might assume that there have been no subsequent modelling papers on the epidemic potential of concurrency since these early works. This is incorrect; subsequent modelling papers have explored the topic in a variety of ways, with similar qualitative conclusions.

In addition, Morris and Kretzschmar's initial work helped clarify how existing modelling methods could not easily or thoroughly explore the concurrency hypothesis, and the past 10 years have seen considerable methodological development to remedy this situation. This work has now begun to pay off, with more recent models being able to incorporate a richer array of empirical data on both behaviour and biology than in the past. The field is now poised to be able to explore the many facets and forms of concurrency in greater depth than ever before. Collectively, all of these provide considerable new support on the modelling side for the concurrency hypothesis.

In this paper, I review the modelling work on concurrency and epidemic potential since the Morris and Kretzschmar papers. I also explain the methodological limitations that explain why the initial progress on this topic was slow. Finally, I explore the methodological developments that have occurred to remedy this, highlight recent work stemming from these, and outline important areas for future modelling research.

## Discussion

### The prevailing framework in the field of epidemic modelling cannot easily or fully address the topic of relational concurrency

The field of epidemic modelling is largely built around a framework known as compartmental modelling, also called mass-action or ordinary differential equation (ODE) modelling. In this framework, individuals are not explicitly represented; only groups of epidemiologically identical individuals ("compartments") are, and their numbers through time are specified with a system of ordinary differential equations. Because individuals are not explicitly represented, neither are their pair-wise relationships or contacts.

The classic versions of these models fall into one of two classes. In the first, relationships are not considered at all; equations simply encode expressions for the *number of contacts *between individuals in each combination of compartments that occur at each time point, where a contact represents a single sex act. There is then a separate term (or terms) for probability of transmission per contact. In the second approach, equations encode expressions for the *number of relationships *between individuals in each combination of compartments that are *initiated *at each time point. These then include a term (or terms) for the infectivity per partnership, which is typically an expression comprising terms for relational duration, number of sex acts per unit time, and infectivity per sex act.

Although this latter approach has the advantage over the former of acknowledging the existence of relationships, the underlying maths includes a subtle assumption: partnerships can only transmit if they are serodiscordant at their outset, not if one member becomes seropositive from outside the relationship during its course. That is, any potential effect of relational concurrency on the epidemiology of HIV is missed by these particular models.

### One approach to explain the consequences of relational concurrency requires dynamic network-based models. These typically require considerably more work than a traditional compartmental model to develop

If the prevailing compartmental modelling framework does not effectively capture concurrency, how does one go about doing so? One answer is to use dynamic network models. Network models include any model in which relationships between pairs of individuals are explicitly represented; they are thus a subset of agent-based or individual-based models, which include all those that represent individuals explicitly.

Dynamic network models-those that model networks over time-have the advantage that they can allow one to consider scenarios that entail differing assumptions about the relative timing and overlap (or lack thereof) in relationships. They traditionally involved a major tradeoff, however. Compartmental modelling's great advantage is its ease of implementation; it requires a familiarity with constructing differential equations-something commonly taught at the college level - and a differential equation software package. The framework also comes with an underlying mathematical theory that has been developed and expanded upon for decades by a large number of researchers.

General theory and tools for dynamic network modelling simply have not existed in the same way. Dynamic network models, and other forms of agent-based models, generally require that one write one's own computer code from scratch. They also immediately open up an enormous number of statistical and logistical issues that compartmental models avoid. Dynamic network models in general, and models of concurrency specifically, entail dependence among relationships. That is, whether or not person *i *forms a relationship with *j *depends on whether each is in other relationships; but whether those other relationships end depends on whether *i *and *j *have partnered.

The end result is that the states of all relationships in the population become recursively dependent. Those well versed in statistics know that dependent data of any kind require far more complex tools to analyze than do independent data, especially when it is the dependence that is of core interest, not merely something to control for.

For their series of concurrency modelling papers, Morris and Kretzschmar developed an elaborate set of computer code to explore a limited set of scenarios. Given the methodological complexities invoked by relational dependence, the code was neatly tailored to those specific scenarios, and not set up to generalize to a much wider range of scenarios. This is not a critique of Morris and Kretzschmar; this is simply what was possible at the time. Indeed, their insights set off multiple lines of research aiming to expand on the work they had done.

### Attempts to extend the compartmental modelling frame to handle relational concurrency yielded novel theoretical approaches, but these were generally cumbersome in practice and did not generate much subsequent applied work on HIV in sub-Saharan Africa

One major advantage of the differential equation modelling approach is that it lends itself to analytical exploration in ways that stochastic simulation methods do not, a quality that has long been highly valued in modelling. Thus, around the same time as, and subsequent to, the initial network modelling work on concurrency, a variety of researchers sought to develop novel ways to extend existing ODE approaches to incorporate representations of couples.

Dietz and colleagues provided early development in pair formation models [2] and then extended these into "triangle" models [3] that kept track of the number of individuals connected to two others in a local network. Although these were major advances, they were still not flexible enough to consider all of concurrency's potential patterns and effects. A variety of new pair formation and moment-closure approaches followed [4-9]. The focus of these papers was largely on method development, so they were in general not concerned with richly parameterizing their model with behavioural and biological data in all of the ways that Sawers and Stillwaggon outline as necessary for a realistic model, although all incorporated some forms of data.

Nevertheless, this line of work confirmed the general hypothesis that concurrency has a strong ability to amplify the transmission of a sexually transmitted infection under a wide variety of scenarios, relative to the same number of relationships occurring in a serially monogamous fashion.

Unfortunately, for all the theoretical richness that this work generated, it did not spawn much subsequent research that used the new methods but with more detailed empirical data. Even the later modelling work done by some of the same research groups that developed these methods has either reverted to more traditional ODE approaches (e.g., Hallett *et al *[10]) or focused on dynamic network models (e.g., Eaton *et al *[11], discussed later). The conventional wisdom is that the methods were highly cumbersome, and not easy to further generalize in ways that would allow them to handle the full complexities of behavioural, biological and demographic data.

There is one example of a recent data-driven model that follows in this vein, however. Johnson and colleagues [12] developed an ODE model that could depict long-term marriage relationships and momentary commercial contacts within or outside of these marriages. They parameterized their model using behavioural data from South Africa, and after exploring a variety of scenarios, concluded that "concurrent partnerships and other non-spousal partnerships are major drivers of the HIV/AIDS epidemic in South Africa" [12].

### In the meanwhile, a variety of network modelling papers have been published that confirm the major sexually transmitted infection (STI) epidemic potential that concurrent relationships generate relative to sequential ones

Although in the past decade, most researchers in the field were seeking ways to explore concurrency within the ODE framework and its extensions, some did opt to build from scratch new network-based models of HIV or STI spread, and parameterize them using biological and behavioural data. Since Morris and Kretzschmar had produced initial analyses of HIV in sub-Saharan Africa, many of these next set of works focused on STIs other than HIV [13-17], or on populations outside Africa [18]. One might assume that for these reasons, it is reasonable that Sawers and Stillwaggon left them out of their review.

However, their argument included two prongs, which they treated separately: that modelling does not convincingly show that concurrency makes a difference to epidemic outcomes; and that data do not show that concurrency is more common in sub-Saharan Africa than elsewhere. These articles are relevant to the first of these two points. Collectively, they added to the growing evidence that under a range of circumstances, concurrency generates considerably larger epidemics than do serial monogamy, even when overall numbers of partners are controlled for.

One work in this line that Sawers and Stillwaggon also ignore focuses specifically on modelling concurrency and HIV in sub-Saharan Africa within an agent-based simulation framework. Leclerc and colleagues [19] sought to reconstruct the dynamics of the HIV epidemic in Zambia, including the age and sex distribution, using demographic and health survey (DHS) data from that country. They built an agent-based model from scratch, and included complex demographic, transmission and behaviour modules; the latter included marriages, non-marital partnerships and commercial sex contacts. Their initial model that most closely reflected DHS data and existing transmissibility estimates yielded an epidemic of 16.6% prevalence for women and 12.0% prevalence for men.

Although they do not specifically provide results for a counterfactual scenario in which concurrent relationships are disallowed, they present a variety of findings that suggest that this population is close to the reproductive threshold (e.g., that R_0 _= 1.95), and that concurrency is crucial for maintaining transmission (e.g., that while 62.5% of HIV-positive females are infected within marriages, only 22.2% of males are). Additional results from this model that explicitly consider the role concurrency plays in maintaining the epidemic will hopefully be forthcoming.

### An enormous effort has been underway for the past decade to develop general tools for data-driven dynamic network modelling, which has only very recently reached the point of allowing us to revisit the concurrency hypothesis with more detail and precision than ever before

Following the Morris and Kretzschmar papers, a large multi-disciplinary group of statisticians and social scientists took the approach of setting out to develop the statistical models and programming tools needed to conduct generalized, dynamic, data-driven social network inference and simulation. This group recognized that the existing tools did not allow for the kinds of rich modelling needed for this and other questions of STI epidemiology to be explored in depth, and felt that the dynamic network framework was the most promising approach to remedy this in the long term. This ambitious research agenda has indeed taken much of the previous decade to fully develop.

At the time, a generalized statistical framework for cross-sectional network estimation and simulation had been proposed [20]. Subsequent years were spent identifying, explaining and overcoming various underlying statistical issues that emerged when implementing this approach in practice [21-23]. Additional computational and algorithmic developments led to the release of a public programming package for cross-sectional versions of these models that has been widely used in a variety of fields http://www.statnetproject.org.

However, additional modelling questions needed to be answered to allow these tools to handle longitudinal data and dynamic network simulations, as well as to use sampled and incomplete data to parameterize, model and simulate complete networks. These latter pieces have only recently been solved [24,25], opening the door for a much broader array of dynamic network modelling investigations.

The first applied HIV epidemiology paper using this approach was recently published [26], appearing well before Sawers and Stillwaggon's article. It demonstrates again that under some conditions, concurrency has dramatically more epidemic potential than does the same amount of sexual contact structured as serial monogamy. The model is parameterized using US data, not African data, and it explores only one feature of epidemic potential (the "reachable path"), given the specific questions it was trying to answer. This is another example of an article that adds to the overall picture of concurrency's potential to drive major HIV epidemics relative to serial monogamy in some settings.

### Recent and pending papers that incorporate our new knowledge of acute infection strengthen the modelling evidence in favour of the concurrency hypothesis

The early work on concurrency did not include different levels of infectivity by stage of infection since this was not clearly understood at the time. Yet the presence of a short period of high infectiousness early in infection should logically enhance the ability for relational concurrency to fuel an epidemic relative to serial monogamy since it allows for people to easily become infected and transmit within a narrow window.

Two additional recent papers re-examine the concurrency hypothesis given the more precise information that has emerged in recent years about per-act transmissibility of HIV during each stage of infection. These confirm that concurrent relationships have a strong ability to amplify the spread of HIV relative to the same number of sequential relationships.

The first of these [11], published by a research group without access to the dynamic *statnet *tools then still in development, chose the time-intensive task of reproducing the original Morris and Kretzschmar model with all new computer code, but with stage-specific transmission probabilities added in. In a reverse of the previous paper [26], they used empirical estimates of infectivity, but a stylized behavioural model, taken from the early Morris and Kretzschmar work. They indeed find that acute infection amplifies the importance of concurrency; for empirical estimates of transmission by stage, the modelled concurrency rates generated epidemics as large as 15% prevalence, whereas simulations with the same numbers of partnerships arranged as serial monogamy or small amounts of concurrency led to the extinction of the HIV epidemic.

The final work [27] is the first to include a model in which both the partnership timing and networks and the biology are fully driven by empirical data, and does so using the *statnet *toolkit. It uses the same stage-specific transmission probabilities [28] that Eaton *et al *[11] do, as well as three other published estimates [29-31], including the empirical data on coital frequency found therein. It incorporates observed levels and patterns of concurrency from a Zimbabwe data set [32], including gender asymmetry, and distinguishes between counts of cohabiting and non-cohabiting partners. The paper finds that the behaviour and network structure observed in 2005, including levels and patterns of concurrency, should generate an epidemic with equilibrium prevalence around 9%. A tiny increase in sexual partnering from the data, from an average of 0.66 partners in the cross-section to 0.70, increases the size of the epidemic to about 14% prevalence.

It is not clear what the equilibrium prevalence would be if people consistently engaged in the behaviours reported in 2005 over a long period of time. What is clear is that rates of sexual behaviour concurrency extremely close to those reported can explain a sizeable generalized HIV epidemic in this population; fallback assumptions about non-sexual transmission are not required. Moreover, like the early work of Morris and the recent Eaton paper, the model shows that the same number of partnerships, with the same durations, occurring sequentially rather than concurrently, eliminates all HIV epidemic potential in this population.

Although the last two articles were not published at the time of Sawers and Stillwaggon's review, they do render obsolete the critiques of the modelling work that might have remained after all of the other work that they left out was accounted for. The crux of their argument-that mathematical models "require unrealistic assumptions about frequency of sexual contact, gender symmetry, levels of concurrency, and per-act transmission rates" - is false. Various models over the past decade have addressed each of these, and one paper now addresses all of them together; collectively, these confirm the crucial role that concurrency can play in driving STI epidemics, and specifically HIV.

### A note on coital frequency

It is worth noting the assumptions about coital frequency that appear within the transmission estimates used by both Eaton *et al *[11] and Goodreau *et al *[27] since this is a specific criticism of the field that Sawers and Stillwaggon raise. The paper that estimated per-act transmission from serodiscordant couples in Rakai, Uganda [31], also included data on coital frequency for those couples by stage of infection. These empirical estimates for coital frequency were then used in Goodreau *et al *[27] for the three of their four transmission models that were built off of published estimates for per-act transmission estimates.

Hollingsworth *et al *[28] reanalyzed the Rakai data, determining that the data did not allow for a clean estimate of per-act transmission probability but only for a per-time-period transmission probability. Both Eaton *et al *[11] and Goodreau *et al *[27] used these estimates as well, the former exclusively, and the latter for their fourth model. In the Hollingsworth framework, there is no explicit estimate for the number of coital acts per time period; however, if all existing estimates are converted into per-month probabilities, it can be seen that the Hollingsworth estimates are in line-about the same in most months, higher in a few and lower in a few - with those in the other three papers that include empirical coital act frequencies. Thus, the implicit coital act frequencies within this scenario are also qualitatively similar to the published estimates.

The modelling papers do assume that coital frequency per relationship is the same regardless of whether an individual is in one or more than one ongoing relationship, which is not always a realistic assumption. This is done to ensure that there is exactly the same number of coital acts across the different scenarios, so that observed epidemic differences do not simply reflect changes in coital acts. Doing otherwise would thus leave the models open to a different critique altogether. See the "future work" discussion in the next section for more on the topic of coital frequency.

### A note on modelling and time

It is also worth clarifying a common misconception about the concurrency modelling literature, one which Sawers and Stillwaggon repeat when they discuss these models in terms of the assumptions they make "[i]n order to generate rapid spread of HIV". Few of these models have the goal of accurately reproducing the rapid rise in prevalence that was observed in parts of sub-Saharan Africa from the 1980 s until the early 2000 s (with Leclerc *et al *[19] as one notable exception). Rather, their goal is to show the equilibrium conditions implied by any particular biobehavioural scenario to see what level of "epidemic potential" such a scenario possesses.

Epidemic modelling theory then allows one to extrapolate from these to other insights, a point that Goodreau *et al *[27] discuss in more detail. Reproducing the original trajectory would require an accurate model of behaviour for the 1970 s and 1980 s, and we simply do not possess the type of egocentric network data needed to represent concurrency from that period. Assuming that models parameterized with behaviour from the 1990 s or 2000 s would reproduce the temporal dynamics of HIV spread in prior periods is akin to assuming that no reductions in partner numbers or in levels of concurrency have occurred anywhere in Africa in the face of the HIV epidemic. This contradicts evidence for behaviour change over observed time periods (e.g., Gregson *et al *[33] and Gouws *et al *[34]), goes against common sense, and denies Africans any agency.

Unfortunately, we will never have the data we need to answer empirically how the initial rise in the epidemic was generated. What we can do now, in the absence of data, is to use dynamic network modelling to identify the conditions under which a major epidemic could unfold in one or two decades. This is an important topic for future research.

### Future work

Now that a general set of modelling tools is available, the recent work is likely only the beginning of a series that further explores specific features or forms of concurrency in more detail. For example, Kretzschmar *et al *[35] pointed out how one form of concurrency (polygyny) can be protective, but only when followed absolutely; what, then, is the level of risk posed under different departures from the absolute? There is more work to be done to determine the conditions under which short-term concurrencies might generate epidemic potential since the modelling work has primarily focused on longer-term concurrencies.

Finally, we know that concurrency's impact can work in at least two different ways: on the one hand, it doubles the number of potential "reachable paths" relative to the same relationships occurring sequentially; on the other hand, it also speeds up the possibility for transmission within existing reachable paths by not requiring the virus to remain "trapped" for some time in a seroconcordant positive monogamous partnership. What is the relative importance of these two amplifying effects?

This is not simply a theoretical question, but actually relates to a specific pattern of concurrency observed in some African settings: the case of circular labour migrants with one partner in each of two locations. The migrant in such a situation will obviously not have regular contact with both partners in the same period. This clearly generates the first of the two amplifying effects (doubling the number of reachable paths), but not necessarily the second one (shortening transmission time on existing paths), depending on the frequency of returns home.

Lurie and his colleagues have used modelling to show the importance of such a system in amplifying disease spread [36], although not in a dynamic network framework; exploring it in this way, so that the results can be directly compared with ongoing work, will help us clarify which of concurrency's two modes of action may be more important overall, or in particular empirical settings. Relaxing the assumption of regular contact within all partnerships is straightforward within the *statnet *network framework, as is relaxing many other assumptions; this should hopefully make future modelling work on concurrency occur more rapidly than it has in the past.

## Conclusions

A solid body of work since Morris and Kretzschmar's early papers strongly confirm the potential for concurrency to play a major role in shaping epidemics of both HIV and other STIs under realistic biological and behavioural scenarios for various sub-Saharan African populations. Sawers and Stillwaggon's argument that the concurrency models of Morris and Kretzschmar only find an effect because of absurd parameters is simply wrong; considerable work since then, and in particular, recent work building off of new behavioural and biological data, and a decade of intervening methodological development, confirms and extends the basic hypothesis. Any remaining questions imply the need for more work on the topic, not less.

Sawers and Stillwaggon end by claiming that the research into relational concurrency as a possible driver of the HIV epidemic aims to "prove Western preconceptions about African sexuality". This is an unfair and unfounded accusation. HIV is a sexually transmitted infection, and a comprehensive research agenda that aims to understand its global disparities will necessarily require exploring sensitive questions about sexual behaviour. Presupposing nefarious intents for those doing so is counterproductive. In contrast to Sawers and Stillwaggon, I end with a call to leave open all promising areas of research in trying to solve one of the world's greatest public health crises.

## Competing interests

The author declares that they have no competing interests.

## Authors' contributions

SMG is responsible for all aspects of the manuscript and has read and approved the final version of this manuscript.
